# The effect of chemotherapeutic agents on epidermal neural crest stem cells

**DOI:** 10.22099/mbrc.2024.49755.1948

**Published:** 2025

**Authors:** Nasim Rahmani-Kukia, Fatemeh Keshavarzi, Mohammad Saied Salehi, Farzaneh Bozorg-Ghalati, Zahra Mojtahedi, Mozhdeh Zamani, Negar Azarpira, Pooneh Mokarram

**Affiliations:** 1 Autophagy Research Center, Shiraz University of Medical Sciences, Shiraz, Iran; 2 Basic and Molecular Epidemiology of Gastrointestinal Disorders Research Center, Research Institute for Gastroenterology and Liver Diseases. Shahid Beheshti University of Medical Sciences, Tehran, Iran; 3 Molecular Medicine Research Center, Hormozgan Health Institute, Hormozgan University of Medical Sciences, Bandar Abbas, Iran; 4 Clinical Neurology Research Center, Shiraz University of Medical Sciences, Shiraz, Iran; 5 Autophagy Research Center, Department of Biochemistry, School of Medicine, Shiraz University of Medical Sciences, Shiraz, Iran; 6 School of Public Health, University of Nevada, Las Vegas, NV 89154, USA; 7 Transplant Research Center, Shiraz University of Medical Sciences, Shiraz, Iran; a The first two authors contributed equally to this work

**Keywords:** Neural Crest Stem Cells, Chemo agents, Autophagy, Unfolded protein responses

## Abstract

Human Epidermal Neural Crest Stem Cells (hEPI-NCSCs), as a transient population of multipotent migratory stem cells can differentiate into multiple types of neural and non-neural cells and tissues in the body. Here, we tried to determine the role of chemo agents in mediating the stress induced pathways like autophagy and unfolded protein responses (UPR), as well as the migratory potential of NCSCs. hEPI-NCSCs were treated with chemo agents including Dithiothreitol [(DTT) 10µM)], Salinomycin (9mM), Ebselen (10mM), 5-Fluorouracil [(5-FU) 8µM] and Cisplatin (6mM) for 72 hours. The reverse transcription-quantitative polymerase chain reaction (RT- qPCR) and scratch wound healing assays were used to assess the effect of chemo agents on NCSCs function. After treatment with DTT, hEPI-NCSCs upregulated the expression of genes related to autophagy and UPR pathways including *LC3*, *P62* and *CHOP*. These genes were also overexpressed when NCSCs were treated with Salinomycin. Reverse results were verified by 5-FU, Ebselen and Cisplatin treatment. Salinomycin and Cisplatin upregulated the expression of *XBP-1*, which down regulated with DTT, 5-FU and Ebselen. Inhibition in migratory capacity of NCSCs was detected following treatment by Salinomycin, 5-FU, Ebselen and Cisplatin. DTT and 5-FU promoted the expression of *BDNF*, while Salinomycin, Cisplatin and Ebselen treatment reduced its expression. During exposition to DTT, the autophagy pathway was activated, implying that autophagy functions as a survival mechanism for deactivating the inhibitory effects of DTT on the migratory capacity of NCSCs. Chemotherapeutic agents like 5-FU and cisplatin exert cytotoxic effects on NCSCs by suppressing autophagy, UPR pathways, and the migratory potential of NCSCs.

## INTRODUCTION

The neural crest stem cells (NCSCs) as a multipotent migratory stem cell population in the dorsal border of neural plate, migrate into embryonic body to differentiate into a variety of cell lineages with considerably unlike actions [1]. These cells have self-renewal and multipotent capabilities [2]. The stimulation of multiple signaling pathways like Wnt, retinoic acid, fibroblast growth factor (FGF), Notch, bone morphogenic protein (BMP) and autophagy induce the activation of several transcription factors that determine the place, where NCSCs form and further progress [3, 4]. Upon epithelial-to-mesenchymal transition, these cells dissociate from their adjacent cells and delaminate from the neuroepithelium to migrate within developing embryo, which cause to undergo different environmental signals [5]. Subsequently, they settle in destination sites to differentiate to multiple cell types containing neurons, glial cells, fibroblasts, intestinal nervous system cells and melanocytes as well as tissues such as bone and cartilage [5, 6]. Dysregulation in NCSCs migration, development and differentiation can result into a wide variety of human congenital neurocristopathy disorders like melanoma, neuroblastoma, cardiovascular and craniofacial defects [7, 8]. Conventional chemo agents are used to suppress cancer cells amplification and tumor progression [9]. However, they can also exert toxic effects on normal cells [10-12]. 

Salinomycin as a K+ ionophore-antibiotic is derived from Streptomyces albus. Although Salinomycin, has been conventionally used as an anti-coccidial drug, recently its anti-cancer effects and functions in overcoming multi-drug resistance of human cancer cells is identified [13, 14]. Ebselen as a seleno-organic drug molecules can mimic the glutathione peroxidase activity and show anticancer effects [15, 16]. Dithiothreitol (DTT) is generally used as reducing agent and inducer of ER stress [17, 18]. Recently, its anti-cancer effects is verified in some experiments [9, 19]. 

The appropriate activity of cells is affected by a variety of physiological and pathological factors both inside the cell (nutrient deprivation, hypoxia and loss of calcium homeostasis) and in its microenvironment, as well as different chemo agents, which can trigger endoplasmic reticulum (ER) stress and subsequently activate unfolded protein responses (UPR) [20]. The UPR pathway restores ER homeostasis by protein synthesis reduction, protein folding capacity enhancement, and degradation pathway activation. If the stressful conditions and the existence of chemo agents persist, autophagy becomes activated as an overall response to a variety of internal and external stress stimuli [21, 22]. Autophagy as a protective process not only recycle the cellular components and remove the aggregated proteins but also quickly supplies energy to sustain cell growth and vital function under stress [23]. Autophagy is also implicated in cellular self-renewal and differentiation as well as embryonic development [24, 25]. Recently, it is suggested that this pathway is important for stem cell differentiation in mesenchymal stem cells, muscle stem cells and neural stem cells [4]. Autophagy initiates with the formation of autophagosomes, which are double-membrane-bound vesicles that fuse with lysosomes during autophagy to promote the degradation of autophagic loads [23].

The effects of chemo agents on function of hEPI-NCSCs have not yet been detected. EPI-NCSCs HFSCs, also known as hair follicle-derived crest stem cells (HFSCs), are reside of embryonic neural crest and located in the bulge area of adult hair follicles and ontologically linked to the central nervous system [2]. Therefore, in this study we try to detect whether chemo agents can affect the function of NCSCs and ER stress related pathways such as UPR and autophagy in these cells.

## MATERIALS AND METHODS

### Materials:

Salinomycin (Batch NO.: BCBS8052V, Sigma), Ebselen (Lot NO.: 087m4019v), 5-Fluorouracil (5-FU, Batch NO.: 201381, Thymoorgan pharmasize GMBH Germany), Dithiothreitol (DTT, CAT Number: D0632, Sigma) and Cisplatin (CAS 15663-27-1, Merck) were prepared in dimethyl sulfoxide (DMSO; Sigma-Aldrich) and diluted with media prior each experiment. α-MEM medium, penicillin-streptomycin and fetal bovine serum (FBS) were purchased from Invitrogen Life Technologies (Gibco, Life Technology).

### Cell viability assay:

 Based on the previously described method [26], hEPI-NCSCs were isolated from the pubic hair skin punches of a 22-year old brain-dead patient, verified by immunostaining, and cultured in alpha-MEM medium containing 10% FBS and 5% CO2 in 37˚C humidified incubator. Then, to determine the suitable doses of Dithiothreitol (DTT), Salinomycin, Ebselen, 5-Fluorouracil (5-FU) and Cisplatin, an MTT assay was performed. Here, 24 hours prior to drug treatment, the cells were plated in 96-well in triplicate. 72 hours after treatment with various concentration of DTT and 5-FU (0, 2, 4, 6, 8, 10, 12, 14 µM), Salinomycin, Ebselen and Cisplatin (0, 2, 4, 6, 8, 10, 12, 14 mM), 20 μl of 5 mg/ ml MTT solution was added to each well and incubated for 4 hours at 37°C to detect the cell viability. After 4 hours, the resulting MTT formazan crystals were solubilized in 100 µl DMSO, and the developed color was measured spectrophotometrically at 570 nm using a microplate reader.

### EPI-NCSCs treatment:

Based on acquired data from the MTT assay, after reaching the third passage with 70% confluency, EPI-NCSCs were trypsinized and seeded in T25 flasks to be treated next day with α-MEM containing Dithiothreitol [(DTT) 10µM)], Salinomycin (9mM), Ebselen (10mM), 5-Fluorouracil [(5-FU) 8µM] and Cisplatin (6 mM) for 72 hours, respectively. DTT is used as reducing agent and inducer of ER stress. Control group was incubated with α-MEM containing FBS 10% plus penicillin/streptomycin 1% in the same culture condition.

### RNA extraction and quantitative reverse transcription-quantitative polymerase chain reaction (qRT- PCR):

Total RNA from hEPI-NCSCs treated groups was extracted using RNA extraction SinaClon Kit following instructions. cDNA was also synthetized employing SinaClon cDNA synthesis kit procedure. Later, the mRNA expression level of brain-derived neurotrophic factor (*BDNF*), *LC3*, *P62*, *XBP-1* and *CHOP* was determined using QuanStudioTM 5 Real-Time PCR system. For real-time PCR, the reaction cycle consisted of 95°C for 15 min, followed by 30 cycles of 95°C for 15 s, and 60 °C for 1 min. Gene expression analysis was performed using 2^-∆∆Ct^ method. In this experiment the *GAPDH* was used as the control for normalization. The primers used in this study were designed by Allele ID v7.5 software (Primer Biosoft International, Palo Alto, CA) and listed in Table 1. 

### Scratch wound healing assay:

hEPI-NCSCs were seeded in 6-well plate to reach proper confluency. A vertical scratch, using a sterile plastic micropipette 10 microliter tip was created on monolayer cells and then cells were treated with interested drugs for 72 hours. The images were captured close the scratch at the beginning and regular intervals during cell migration. The images were compared to assess the rate of cell migration.

### Statistical analysis:

Data were given as mean ± SD of three replication (n=3). Statistical information analysis is reported via GraphPad Prism using one-way ANOVA and the Tukey post-hoc test. p<0.05 is regarded as statistically significant. In all figures *, **, and *** means that p-values were <0.05, <0.01, and <0.001, respectively.

## Results

**Figure 1:**
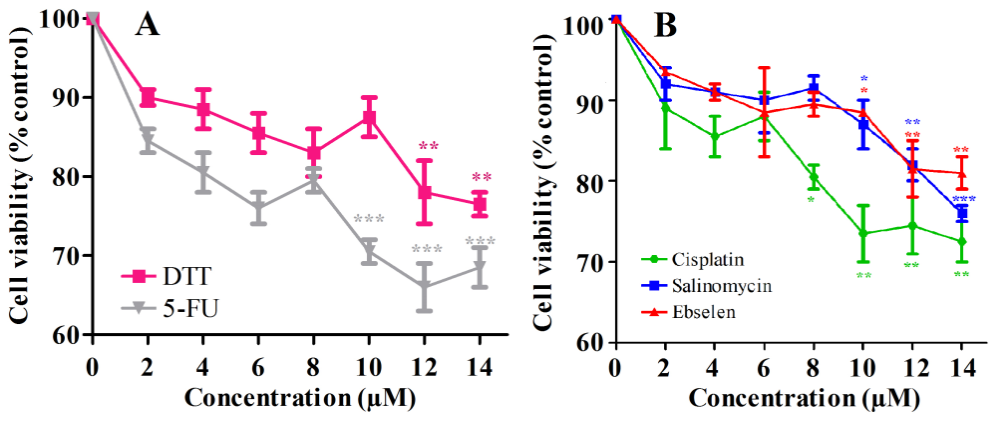
Effect of chemo agents on viability of hEPI-NCSCs. The cells were treated with different doses of DTT and 5-FU (0, 2, 4, 6, 8, 10, 12, 14 µM) (**A**), and Salinomycin, Ebselen and Cisplatin (0, 2, 4, 6, 8, 10, 12, 14 mM) (**B**) for 72 hours.

The MTT assay was employed to determine the safe and proper concentration of interested drugs for EPI-NCSCs treatment. The acquired data showed the viability of EPI-NCSCs following treatment with 10µM DTT, 9mM Salinomycin, 10mM Ebselen, 8µM 5-FU and 6mM Cisplatin to be very close to the control group, which were suitable for our scope. Accordingly, drugs at these concentrations were chosen for further evaluation of their impacts on the expression level of various autophagy and UPR transcript and EPI-NCSCs' migratory behavior. Figure 1 shows the results of MTT assay following 72 hours EPI-NCSCs treatment with various doses of drugs.

**Table 1:** The Primer sequences of used genes

**Genes**	**Forward primers (5'-3')**	**Reverse primers (5'-3')**
*LC3 *	AACGGGCTGTGTGAGAAAAC	AGTGAGGACTTTGGGTGTGG
*P62*	AATCAGCTTCTGGTCCATCG	TTCTTTTCCCTCCGTGCTC
*BDNF*	TGTGCCGGGTGTGTAATC	CTCACCTGGTGGAACTGG
*Chop*	GCTCTGATTGACCGAATGG	TTCTGGGAAAGGTGGGTAG
*XBP-1*	TGCTGAGTCCGCAGCAGGTG	GCTGGCAGGCTCTGGGGAAG
*GAPDH*	CGACCACTTTGTCAAGCTCA	AGGGGTCTACATGGCAACTG

**Figure 2:**
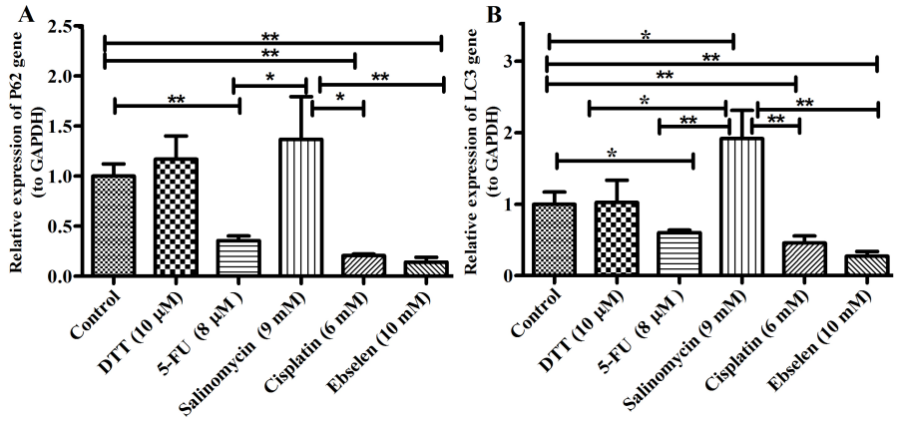
Autophagy genes expression *P62* (A) and *LC3* (B) in chemo agents treated groups of EPI-NCSCs.

To measure the effect of chemo agents on autophagy pathway, hEPI-NCSCs were treated with DTT, Salinomycin, Ebselen, 5-FU and Cisplatin for 72 hours. Then, the gene expression of key autophagy markers like *P62* and *LC3* was determined, using qRT-PCR analysis. As shown in Figure 2, 72 hours treatment of EPI-NCSCs with 8 µM 5-FU, 6 mM Cisplatin and 10 mM Ebselen could decrease the expression level of *LC3* by 60%, 45% and 27%, respectively. While, 9 mM Salinomycin increased the expression level of *LC3* by 1.9 fold compared to control cells. In addition 10 µM DTT had not significant effect on *LC3* expression in NCSCs. Besides, the P62 mRNA level showed a significant reduction in EPI-NCSCs with 8 µM 5-FU, 6 mM Cisplatin and 10 mM Ebselen treatment following 72 hours (35%, 20%, and 13%, respectively). However, with 10 µM DTT and 9 mM Salinomycin treatment, the *P62* expression level displayed a non considerable enhancement (Fig. 2). 

**Figure 3:**
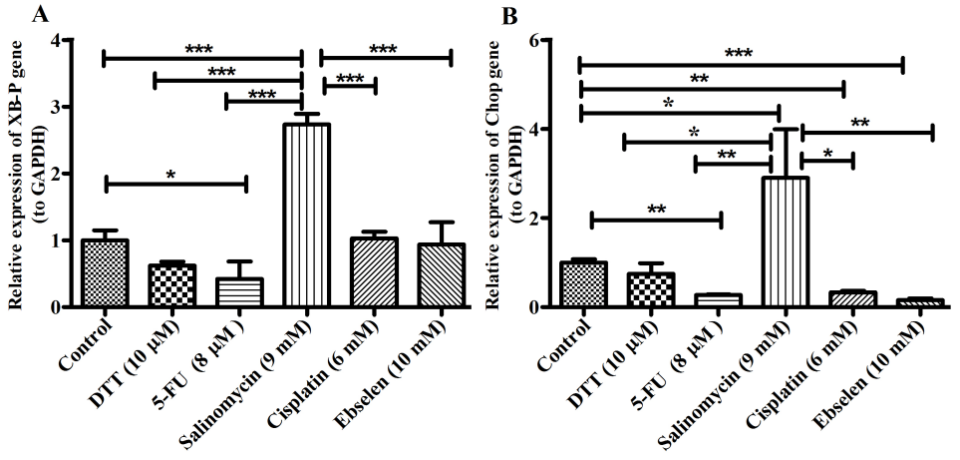
Genes expression level of *XBP-1* (A) and *CHOP* (B) in chemo agents treated groups of EPI-NCSCs.

Quantitative RT-PCR was used to verify the relative changes in the *XBP-1* and *CHOP* mRNA expression level, after treatment the hEPI-NCSCs with chemo agents. The relative mRNA expression level of *XBP-1*, did not significantly increase following 72 hours treatment with 6 mM Cisplatin and 10 mM Ebselen compared to the control cells (Fig. 3). However, *XBP-1* mRNA expression level after 72 hours treatment with 9 mM Salinomycin was remarkably upregulated (2.7 fold). In addition, treatment of NCSCs with 8µM 5-FU significantly reduced the expression level of *XBP- 1* compared with control cells (40%). While, 10µM DTT had not significant reduction effect on *XBP- 1* expression level.

A significant enhancement in the *CHOP* expression level of cells-treated with 9 mM Salinomycin was seen (2.9 fold). However, treatment of EPI-NCSCs with 8 µM 5-FU (27%), 6 mM Cisplatin (33%) and 10 mM Ebselen (13%) decreased *CHOP* mRNA expression level following 72 hours. In addition, *CHOP* mRNA expression level after 72 hours treatment with 10 µM DTT was not remarkably down-regulated (Fig. 3).

**Figure 4:**
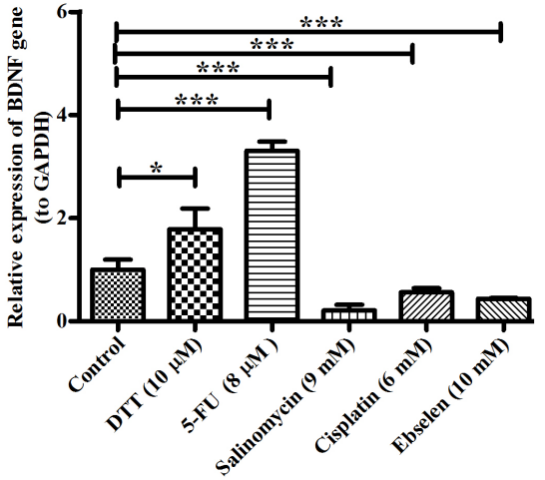
*BDNF* gene expression level in chemo agents treated groups of EPI-NCSCs.

hEPI-NCSCs were treated with chemo agents for 72 hours, and then the *BDNF* mRNA expression level was measured to discover whether chemo agents can affect the *BDNF *expression. BDNF as a member of neurotrophin family is involved in survival, growth, differentiation and plasticity of neural cells in neural system. It can also exert neuroprotective impacts under adverse circumstances. *BDNF* was overexpressed by 10 µM DTT (2.0 fold), and 8 µM 5-FU (3.3 fold) treatments after 72 hours (Fig. 4). However, following 72 hours *BDNF* mRNA expression level was significantly down-regulated with 9 mM Salinomycin (21%), 6 mM Cisplatin (50%), and 10 mM Ebselen (43%) treatments in compare to control cells.

**Figure 5:**
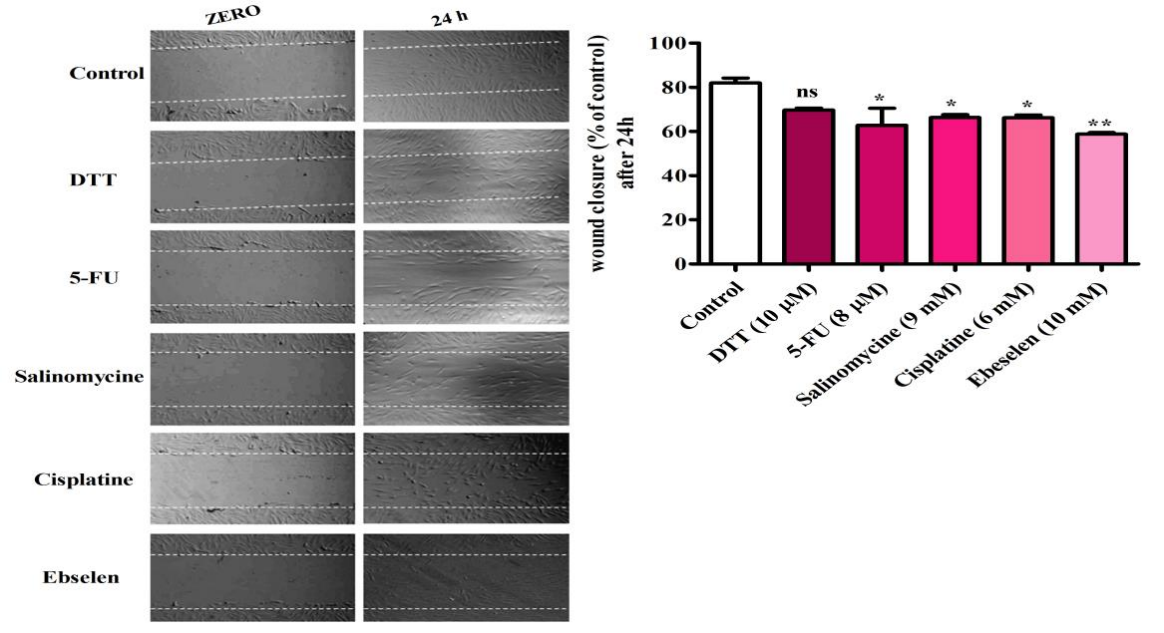
Effect of chemo agents on migration of EPI-NCSCs. The scratch wound was created using 10 µl sterile pipette in 70% confluency of EPI-NCSCs. Media containing certain concentration of each drug was added to defined wells, and the images were taken at 0 and 24 hours by an inverted microscope (×40 magnification). The lines show the area where the scratch wound was created. The scratch wound assay was performed in triplicate wells for each concentration.

 To found the effect of chemo agents on migratory capacity of NCSCs, scratch assay was carried following treatment the cells with these agents. According to the results (Fig. 5), the migratory capacity of hEPI-NCSCs decreases by 8 µM 5-FU, 9 mM Salinomycin and 6 mM Cisplatin treatments (P<0.05). Our data in Figure 5 demonstrated that 10 mM Ebselen treatment more efficiently enhances the migration of NCSCs compared to control cells (P<0.01). However, 10 µM DTT exerted non-significant reduction on migratory ability of hEPI-NCSCs.

## DISCUSSION

NCSCs have self-renewal and multipotent features, which can differentiate into multiple cells types with various functions. The activation of several signaling pathways like autophagy can trigger the activation of some transcription factors involved in NCSCs differentiation, which can be affected by environmental signals [3, 27-29]. Therefore, the aim of our study was to evaluate the impact of chemo agents on NCSCs regarding ER stress induced pathway genes. Our results revealed that chemo agents like 5-FU, Cisplatin and Ebselen downregulate the mRNA expression level of autophagy and UPR factors as well as migratory ability in NCSCs.

 Autophagy and UPR as key pathways involved in intracellular metabolic hemostasis and adaptive defense against stress, determine cells fate [30]. During autophagy process, misfolded intracellular proteins or damage organelles become encapsulated with double-membrane vesicles to be degraded by lysosomes. Stress conditions like starvation, energy-loss, chemotherapeutic agents and hypoxia are main trigger of autophagy [31]. As a result, autophagy by mediating cellular survival through inhibiting cellular damage and regulating organelle recycling, play crucial role in physiological condition. Dysfunction in this process can cause the development of various diseases such as cancer, neurodegenerative, inflammatory and infection diseases [32]. 

ER controls the quality of secretory proteins and accumulation of mis- and un-folded proteins triggers ER stress and UPR pathway. Subsequently, a series of ER transmembrane proteins including PERK, ATF6 and IRE1 become activated to reduce the load of proteins in ER, overexpress the chaperones involved in protein folding and enhance ER associated degradation for misfold protein degradation [33]. Chemotherapeutic agents are widely developed to improve the survival rate of cancer patients [34]. Antimetabolites as a class of efficient chemo agents interfere with critical biochemical processes. Regardless of their widespread application as chemotherapy in cancer treatment, therapy with these antimetabolites can lead to numerous side effects like neurotoxicity and developmental toxicity [35, 36]. In this regard, our results revealed that 5-FU, Cisplatin and Ebselen treatment inhibit the migratory capacity of hEPI-NCSCs by downregulating the key-autophagy and UPR markers gene level. The activation of autophagy pathway can affect the migration, development and differentiation capacity of NCSCs [4, 24, 27, 29]. The suppressive effect of 5-FU on the expression of some neural differentiation marker genes is demonstrated [37]; however, variation in autophagy markers was not determined.

In the context of DTT, the activation of key autophagy genes in hEPI-NCSCs appears as a potent approach to reduce chemo agent effects. Like an investigation that identified 5-FU and oxaliplatin treatment mediate the core-autophagy protein level in chemoresistance colorectal cancer cells [38]. In addition, autophagy is investigated as an escape mechanism through 5-FU treatment in colorectal cancer cells [39-41]. However, our results didn’t show the activation of autophagy genes in hEPI-NCSCs during 5-FU treatment. 

## Authors’ Contribution:

NR-K and FK did all experiments, statistical analysis, figure preparation, and prepared the first draft of the manuscript. FBG and ZM contributed to data analysis and figure preparation. MSS isolated and cultured the stem cells. PM, MZ, NA supervised the project, did a final proof of the manuscript, and provided financial support for the project. All authors have read and agreed to the published version of the manuscript.
